# Decreasing urgent repeat cesarean sections by offering complimentary ultrasounds and consultation in rural Burundi: The zigama mama project

**DOI:** 10.3389/fgwh.2023.1053541

**Published:** 2023-02-28

**Authors:** Eric McLaughlin, Matthew Nagy, Jean-Bosco Magorwa, Gilbert Kibinakanwa, Rachel McLaughlin

**Affiliations:** ^1^ Frank Ogden School of Medicine, Hope Africa University, Bujumbura, Burundi; ^2^ Department of Education, Kibuye Hope Hospital, Kibuye, Burundi; ^3^ Ministry of Public Health and Fight Against AIDS, Kibuye Health District, Kibuye, Burundi

**Keywords:** cesarean section, obstetrical ultrasound, Burundi, health care access, health district, urgent cesarean section, repeat cesarean section

## Abstract

**Objective:**

Repeat urgent cesarean sections (CS) carry an increased risk of severe maternal outcomes. As CS increase in sub-Saharan Africa, creative strategies are necessary to reduce the rate of urgent repeat CS. The Zigama-Mama Project in rural Burundi uses complimentary ultrasounds to create a clinical touchpoint to counsel women with a prior CS for a hospital-based delivery.

**Methods:**

From July 2019 to June 2020, complimentary ultrasounds were offered to all antenatal patients with prior CS, along with counseling for monitored trial of labor after cesarean (TOLAC) or scheduled repeat CS. Community engagement and feedback from district health centers were evaluated.

**Results:**

In total, 500 women with a prior CS presented for a complimentary ultrasound. During the intervention year, a relative and absolute reduction in urgent repeat CS (baseline: *n *= 114 {70.8%}, intervention: *n* = 97{49.7%}, *p* < 0.001) was observed, with no significant change in maternal mortality or ruptured uteri. All health center personnel agreed the project improved their confidence in referring women with prior CS.

**Conclusion:**

Offering complimentary ultrasounds as a clinical touchpoint for scheduling a monitored delivery or CS for women at high risk for delivery complication may be an affordable and creative strategy to care for women with previous CS during subsequent deliveries.

## Introduction

Access to safe and timely cesarean sections (CS) is a foundational aspect of reducing maternal and neonatal morbidity and mortality worldwide (1). Africa has the lowest rate of CS worldwide at 7.3%, and Burundi has a CS rate of 4.0%, both of which are well below the 10% rate over which the World Health Organization (WHO) has noted a lack of population benefit (2–4). As the rate of CS has recently been increasing in Burundi and other sub saharan African countries, issues related to deliveries with scarred uteri and access to safe care remain paramount (5, 6).

Though discussions regarding trial of labor after cesarean (TOLAC) vs. elective repeat CS in rural sub-Saharan Africa continue, it is clear that deliveries after CS carry an increased risk (1, 4, 7–9). WHO data show that women with more than one prior CS have twice the rate of severe maternal outcomes due to failed TOLAC and subsequent urgent C-section (10), creating a need for quick high-quality interventions for these women (8). While TOLAC may be safe for women at otherwise low risk of complication, elective repeat CS is safer for women at high risk for complication (11, 12). Moreover, regardless of risk status, TOLAC is safest undertaken in a monitored healthcare center with CS capabilities if necessary (4).

All urgent CS are associated with increased risk compared to elective CS, especially in LMICs, with this risk elevated further in the second stage of labor (1, 4, 13). WHO data demonstrate that urgent CS is associated with more fresh stillbirths, neonatal deaths, and severe neonatal morbidity compared to elective CS, findings that were more pronounced in women with a uterine scar (14). Another study in LMICs in 2019 shows that urgent CS (compared to scheduled) was associated with twice higher maternal mortality, five times more perinatal deaths, ten times more postpartum infections, as well as increased postpartum hemorrhage and anemia (1). Despite these risks, a 2019 study of 22 African countries found that 78% of CS were urgent, which is likely one factor leading to the 50-times higher maternal mortality for CS in Africa compared to high-income countries (12).

As access to a hospital with CS capability in many sub-Saharan African countries is scarce, there have been various efforts aimed at improving this access for women with high risk pregnancies (15). Uganda, for instance, implemented a 24 h ambulance service to transport laboring women to the hospital, and South Sudan initiated a hospital referral system offering free transportation and hospital care for qualifying patients (16, 17). While these interventions increased CS rates, the women who gained access were those already requiring urgent, non-scheduled, CS due to complication. As such, interventions aimed at preventing urgent CS are particularly desirable in regions with sparse hospital access. The Burundian Ministry of Public Health and Fight Against AIDS has recommended that all women with scarred uteri deliver in a hospital setting with physician management and a capacity for CS, instead of an outlying health center with less infrastructure (18).

Over the last decade, Kibuye Hope Hospital (KHH), a rural district hospital in Burundi, continued to note a high rate of urgent repeat CS, leading to the development of the Zigama-Mama quality improvement intervention (‘Protect the Mama’ in the Kirundi language). This intervention aimed to reduce the rate of urgent repeat CS for women in the Kibuye Health District through district health center training and engagement, and increasing access to hospital-based TOLAC or scheduled repeat CS. The secondary objective of the project was to strengthen collaboration and cooperation between KHH, the district hospital, and the outlying district health centers.

## Methods

*Program area.* Kibuye Hope Hospital (KHH) is located in Gitega province near the geographic center of Burundi in a rural area approximately a 3 h drive from the largest city, Bujumbura. KHH is the only hospital (and only site to perform c-sections) for the Kibuye Health District which comprises a geographic area of 534 km^2^ and serves over 200,000 people. In addition to KHH, Kibuye Health District has 18 community health centers staffed by nurses who perform antenatal visits and vaginal deliveries. KHH serves as the primary referral hospital for all health centers in the district.

*Program description and population.* The Zigama-Mama Project debuted with a one-day event at KHH including hospital staff and two representatives from each of the 18 district health centers. Two educational programs were offered to participants (*Helping Babies Breathe* and *Helping Mothers Survive*) and the program and its rationale were explained (19). The intervention consisted of *p*regnant women with a history of CS (irrespective of gestational age) who presented for antenatal care to any health facility in the district being offered a coupon for a complimentary ultrasound at KHH. Every woman who presented with this coupon was scanned, and identified concerns were followed up. As per hospital guidelines prior to intervention implementation, women with one prior CS were counseled to TOLAC at the hospital with the exception of previous uterine rupture, myomectomy, inter-delivery interval of less than 24 months, current malpresentation, current placenta previa, or multiple gestation. Otherwise, women with multiple prior CS and/or presence of the above risk factors, were scheduled for CS around 39 weeks gestation.

During the 12 months of the program, a delegation from KHH, including an obstetrician, visited each of the district health centers in order to observe the facility directly, reinforce the protocol, and answer questions. At the end of 12 months, a representative from each health center attended a debrief session at KHH to hear a summary of the program and its outcomes and to give feedback on their experience. The Zigama-Mama project was designed and implemented as a local quality improvement project, as such no informed consent was obtained at the time of the intervention. All patient data were extracted from routine patient paper charts and deidentified for statistical evaluation. The protocol was evaluated and subsequently approved by the Kibuye Hope Hospital Institutional Review Board.

### Program evaluation

*Baseline* vs. *Intervention* A pre-post design was utilized to evaluate the effect of the intervention. Paper charts of women undergoing CS at KHH were evaluated every three months for a baseline period from 1 April 2017 to 31 March 2018. During the 12-month intervention period from 1 July 2019 to 30 June 2020, charts of all women receiving a CS were evaluated every three months. CS indication (urgent vs. elective) and maternal outcomes were collected, cross-referenced and verified with the hospital operative register for all women included.

*CS Rate Evaluation* The number of total deliveries (vaginal + CS) and CS completed in the district were recorded by the local ministry of health, which includes cumulative information from all 18 health centers and the hospital. The number of women with prior CS were estimated based on the calculated CS rate during the baseline year, with the assumption that the CS rate has been relatively stable in the years leading up to the intervention.

*Health Center Feedback Questionnaire:* Representatives of all 18 health centers completed a questionnaire assessing their health center's experience with Zigama Mama. The survey was adapted from the Acceptability of Intervention Measure, Intervention Appropriateness Measure, and Feasibility of Interventions Measure and translated into French by Zigama Mama staff (20). Health center representatives responded to a total of 13 statements on a 5 point likert scale from ‘Strongly Disagree’ to ‘Strongly Agree’, followed by an open-ended question about improvements they would like to see in the future.

*Power analysis.* As a quality improvement project, we did not have a specific enrollment target, rather all women who met criteria were eligible for the intervention. Assuming the number of C-sections performed on women with scarred uteri during the intervention period would be at least equal to the baseline year (164 women), we had 80% power to detect an effect size of 0.15 at an ɑ of 0.05.

*Data analysis.* Comparisons between baseline and intervention data were evaluated by chi-squared analysis or fisher exact test for categorical data (frequency and percentage) and *t*-test (mean ± standard deviation) for numeric data. A logistic regression was conducted, with age adjustment, to evaluate the odds of urgent CS during the intervention year vs. baseline year for women within the district. Odds ratio, *p*-value and 95% confidence interval are reported. CS rate for all women in the district and for women with prior CS relative to all women in the district were evaluated with chi-square test. Health center feedback data was compiled and reported as frequency and percentages response from each of the 18 health centers on a likert scale ranging from strongly disagree to strongly agree. ɑ for all statistical tests was set at 0.05. All analyses were conducted on R 4.2.0 (R Foundation, Vienna, Austria).

## Results

*Population participation.* During the 12-month intervention, exactly 500 women with prior uterine scar presented to KHH with a Zigama Mama coupon given by a district health center and received a complimentary ultrasound and consultation. The average gestational age for women at time of presentation for ultrasound at KHH was 25.9 ± 8.4 weeks. All 18 health centers participated and had at least one patient arrive for an ultrasound.

**Figure 1:**
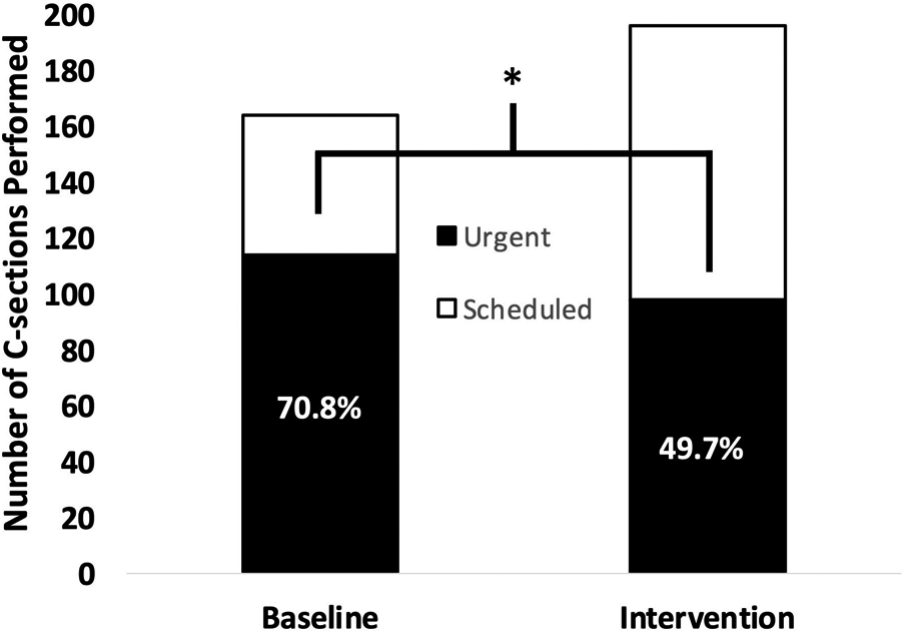
Differences in urgent vs. scheduled repeat cesarean section in baseline and intervention periods for women within the Kibuye district. *=*p *< 0.001.

*Comparison of baseline and intervention periods.* Overall, the total number of repeat CS on women within the Kibuye district increased from 164 in the baseline period to 196 in the intervention period (Table 1). The absolute and relative number of repeat urgent CS decreased from 114 (70.8%) in the baseline period to 97 (49.7%) in the intervention year (*p* < 0.001, Figure 1). The decrease in urgent CS was observed in an age group significantly older in the intervention period (31.25 ± 4.9 years) compared to the baseline period (29.84 years, *p *= 0.007). Accounting for age, women from within the district (intervention received) who received a CS during the intervention year were 2.32 times less likely for it to be urgent compared to women during the baseline year (*p *< 0.001, Table 2). If we suppose an unchanged rate of urgent repeat CS (70.8%) from the baseline to intervention year, we would have anticipated 139 urgent repeat CS during the intervention year. Instead, we observed only 97 urgent repeat CS and thus 42 urgent repeat CS were prevented by offering ultrasound and consultation to all women with prior scars, and performing 500 ultrasounds, which would equate to the number of ultrasounds needed to prevent one urgent CS to be twelve. There was no significant difference in secondary outcomes of maternal death (base: 0.6%, int: 0%, *p* = 0.93), infant mortality (base: 1.2%, int: 3.6%, *p *= 0.28), or frequency of uterine rupture (base: 1.2%, int: 3.6%, *p *= 0.27) (Table 1).

**Table 1:** Additional outcomes between the baseline and intervention periods.

	Baseline Year	Intervention Year	*p*
**Number of C-sections**	164	196	
**Age (mean (SD))**	29.84 (4.83)	31.25 (4.89)	0.007
**Urgent C-section (%)**	114 (70.8)	97 (49.7)	<0.001
**Live Birth (%)**	162 (98.8)	189 (96.4)	0.278
**Mother Survived (%)**	163 (99.4)	196 (100.0)	0.929
**Ruptured Uterus (%)**	2 (1.2)	7 (3.6)	0.271

**Table 2:** Logistic regression exploring the odds of having received an urgent repeat C-section based on whether the delivery occurred during the baseline year or intervention year. The model was adjusted for age. n= 355, * = p<0.05

Predictor	Coefficient (odds)	p-value	95% CI
Age	0.96	0.04*	0.91 - 0.99
Intervention year (vs baseline)	0.43	<0.001*	0.27 - 0.67

While the number of women with a prior scar who delivered (CS + vaginal) in the district health centers was not known, the overall number of deliveries and CS in the district for all women (with/without prior scar) was available from the district ministry of health. The number of CS during one month of the intervention year were missing and were imputed as the mean of the other 11 months. During the baseline year, there were 450 total CS out of 8,553 deliveries (CS rate of 5.2%) and during the intervention year there were 613 total CS out of 9,196 deliveries (CS rate 6.6%, *p* < 0.01). Assuming the number of women receiving repeat CS is proportional to the CS rate during the baseline year (5.2%), the overall repeat CS rate was 37% (196/8553*0.052) in the baseline period and 41% (164/(9196*0.052)) in the intervention period (*p* = 0.2), and the urgent repeat CS rate was 26% (114/(114 + 8103)) in the baseline period and 20% (97/(97 + 8634)) in the intervention period (*p* = 0.06).

**Figure 2:**
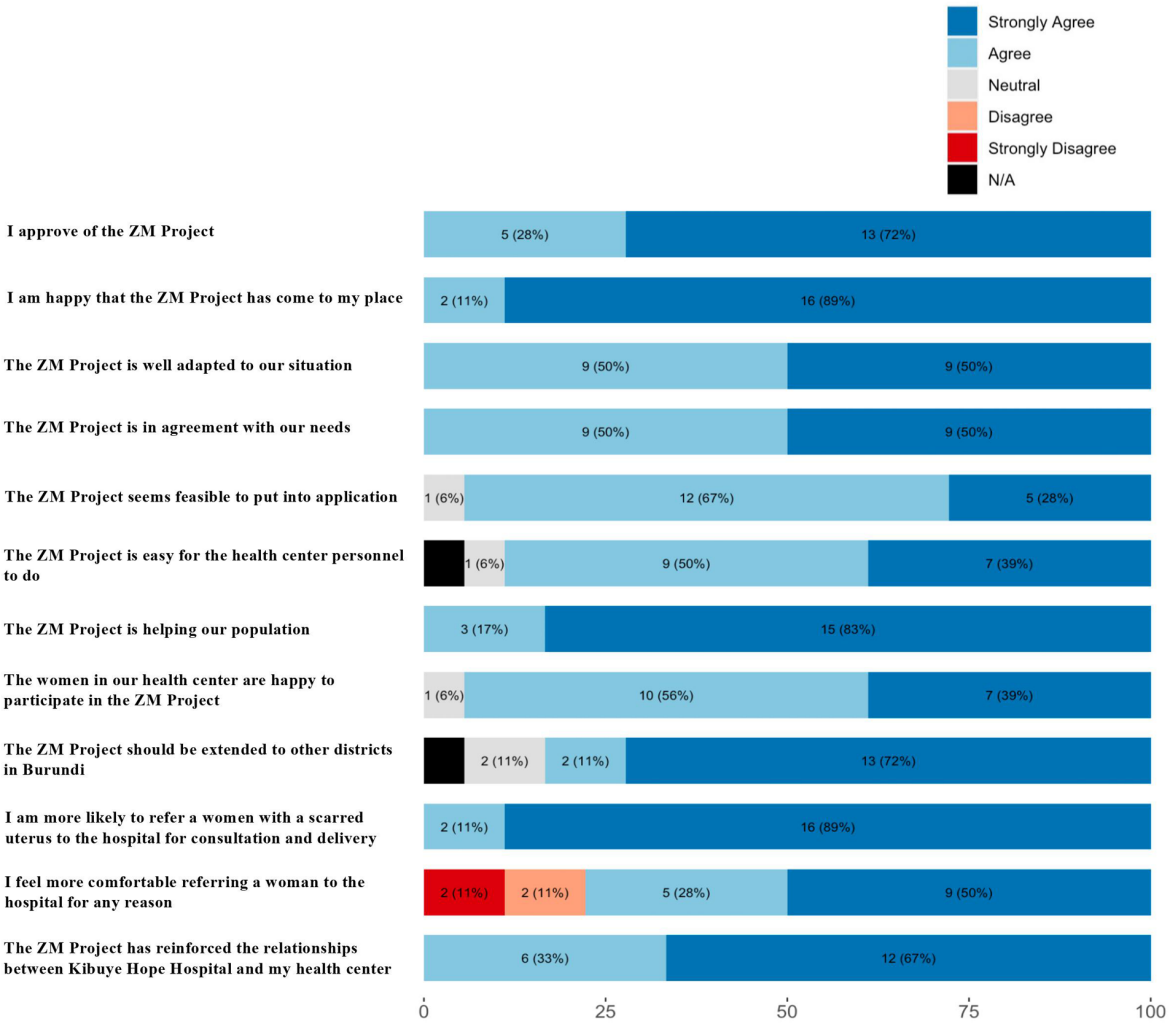
Results of feedback from personnel of the 18 health centers in kibuye health district regarding the zigama mama project., *n* (%). Each of the twelve questions were asked and evaluated by a representative of each of the 18 health centers on a 5 point Likert scale (strongly disagree to strongly agree). Data are presented as frequency and percentage of response to each question.

*Feedback from health centers.* Overall, feedback from health personnel regarding the program was broadly positive (Figure 2). All health center representatives agreed or strongly agreed that the Zigama-Mama project reinforced the relationship between KHH and their health center, and that they are more likely to refer a woman with a scarred uterus to the hospital for consultation and delivery as a result of the program. A large majority of health centers agreed or strongly agreed that they feel more comfortable referring a woman to the hospital for any reason. In a final open-ended question during the feedback questionnaire, many health center personnel noted the major obstacle for women's participation being transport to the hospital where the complimentary ultrasound was performed.

## Discussion

The primary objective of the Zigama-Mama project was to decrease the rate of urgent repeat CS through offering complimentary ultrasound evaluation and counseling for monitored TOLAC or scheduled repeat CS as appropriate. Overall, the Zigama Mama project met its primary objective as shown by an absolute and relative reduction of urgent repeat CS in the Kibuye Health District during the intervention year. Moreover, we did not observe a significant increase in repeat CS rate between the baseline and intervention year, and the proportion of repeat urgent CS relative to all C-sections and all deliveries was lower during the intervention year. No differences were observed in secondary outcomes such as maternal/infant mortality, which may be due to the relatively short time period paired with the overall rarity of these events. Nonetheless, the well-established benefits of avoiding urgent repeat CS (1) suggests the observed decrease in urgent repeat CS may be a useful proxy for decreasing various negative patient-centered outcomes.

There are multiple possible reasons for why offering women with prior CS an ultrasound and consultation resulted in a decrease in urgent C-sections. In many cases, women may not be aware of the increased risk of complication with TOLAC, and ultrasound is not routinely offered at district health centers. As such, being provided a coupon to present at KHH for consultation allows for opportunity for patient education and the identification of high-risk features in the patient's history. The implementation of the ultrasound adds an additional measure to identify high-risk presentations not otherwise apparent based on history or physical examination such as placenta previa, malpresentation, and multiple gestation. Moreover, the local health center implementation may have improved reach as the staff of these centers are trusted members of the local community.

The second objective of the Zigama-Mama project was to strengthen the general sense of collaboration between the district hospital and its health centers. Close collaboration with trusted community healthcare providers has been shown to be a key influence in preventive care in sub Saharan Africa. In Ghana, for example, partnership between modern health systems and traditional birth attendants located in communities farther from the hospital has been shown to increase trust among community members in seeking modern health care (21). Feedback from all 18 health centers strongly suggests that Zigama Mama contributed toward increased community partnerships, with leadership from almost all health centers agreeing or strongly agreeing to every statement. While every health center agreed that their likelihood to refer women with a scarred uterus for hospital consultation was increased, a few health centers, however, disagreed that the program created a spillover effect of increasing their comfort in referring to the hospital for any reason. This suggests that additional interventions may be necessary for non-obstetric indications where hospital referral may be valuable to patient care and safety. Nonetheless, the overwhelming response was that the project was viewed very favorably, suggesting that the Zigama-Mama project offers a feasible model for increasing local health centers' capacity and trust in referring women with high-risk pregnancies for further evaluation.

One issue noted by multiple health centers was the lack of transportation available in the community, limiting the ability for many mothers to take advantage of the complimentary ultrasound at the hospital. This is consistent with experiences of patients across rural sub-Saharan Africa. In rural Malawi, a cluster randomised national household survey found that less than 50% of individuals have access to public transportation, and more than one third noted that even if they required hospital transport, they could not afford the ride (22). In Uganda, various studies have highlighted the role of transportation barriers in contributing to decreased healthcare access, showing worse HIV outcomes for those unable to reliably attend clinic visits (23). As such, investment into affordable public transport infrastructure is a systemic change that would likely meaningfully decrease barriers to safe care for women with prior CS.

Despite the favorable findings, the Zigama-Mama project faced several limitations. First, this project was designed as a pre-post quality improvement project and thus numerous variables were not collected in the historical baseline period, and thus could not be included in this analysis. Second, given the relatively short duration of the intervention (twelve months), there may not have been appropriate statistical power to detect differences in rarer secondary outcomes such as maternal mortality or frequency of uterine rupture. Third, while the intervention could evaluate outcomes of all women who obtained a CS, district health center records regarding women who delivered vaginally with prior CS at district health centers or at home were not accessible limiting our ability to know the true CS rate in this population. Nonetheless, our estimation of number of women with prior scar based on ministry of health data and the baseline CS rate likely offers a conservative estimate of number of women with repeat CS in the intervention year, as the number of women delivering with a prior scar was unlikely to have decreased during the intervention year. Despite these limitations, the Zigama Mama intervention had several strengths. First it took a community partnership approach, coordinating with trusted local health care providers to deliver initial care and referral of patients to the hospital. As the primary referral center for the district, the hospital records likely included a large majority of CS (both urgent and elective) that occurred for women in the designated intervention area. The program model is sustainable and scalable as there are minimal upfront and continuity costs for this intervention, as ultrasound is cheap and accessible, and it is limited only to women with the specific indication of a prior CS. Finally, the evaluation of the program included an in-person feedback session to assess local providers perceptions and address challenges and concerns that arose, allowing for iterative improvement and barrier identification.

Globally, as access to CS increases, creative strategies and systems must be investigated to ensure that complications in women with prior CS are mitigated, especially in areas where rapid access to hospital services is limited. Moreover, within the Kibuye district (and likely other districts), novel solutions to augment patient transportation should be explored and piloted in order to increase access both for advanced pregnancy care as well as other diverse health care needs. Nonetheless, the results of the Zigama-Mama project suggest that offering complimentary ultrasounds as a way to integrate women into a system of monitored TOLAC and elective repeat CS when necessary is one such strategy to reduce the rate of urgent repeat CS and improve health center-hospital relationships in rural Burundi.

## Data Availability

The raw data supporting the conclusions of this article will be made available by the authors, without undue reservation.
